# Lifestyle behaviors, obesity, and perceived health among men with and without a diagnosis of prostate cancer: A population-based, cross-sectional study

**DOI:** 10.1186/1471-2458-8-23

**Published:** 2008-01-22

**Authors:** Laura Q Rogers, Kerry S Courneya, Rammarayan Paragi-Gururaja, Stephen J Markwell, Remi Imeokparia

**Affiliations:** 1 Department of Medicine, SIU School of Medicine, Springfield, IL, USA; 2 Faculty of Physical Education and Recreation, University of Alberta, Edmonton, Alberta, CA, USA; 3 Department of Public Health, University of Illinois Springfield, Springfield, IL, USA; 4 Division of Statistics and Research Consulting, SIU School of Medicine, Springfield, IL, USA

## Abstract

**Background:**

A better understanding of how prostate cancer survivors differ from men without prostate cancer and whether these potential differences vary across demographic subgroups will help to focus and prioritize future public health interventions for improving the health and well-being of prostate cancer survivors. Therefore, our study aims were to compare lifestyle behaviors, body mass index (BMI), and perceived health in men with and without a diagnosis of prostate cancer in a national, population-based sample and to explore whether these comparisons differ for demographic subgroups.

**Methods:**

In a cross-sectional study, men aged ≥ 40 were identified from the Behavioral Risk Factor Surveillance System (BRFSS) 2002 data (n = 63,662). Respondents reporting history of prostate cancer (n = 2,524) were compared with non prostate cancer controls (n = 61,138) with regard to daily fruit and vegetable servings (FVPD), smoking, alcohol, sedentary behavior, BMI, and perceived health. Multivariable logistic regression calculated adjusted odds ratios (OR) and 95% confidence intervals (CI) for the entire sample and for age, race, education, and urbanicity subgroups.

**Results:**

Men with prostate cancer did not differ from men without prostate cancer with regard to smoking, alcohol, sedentary behavior, and obesity but were more likely to consume ≥ 5 FVPD (OR, 95% CI: 1.30, 1.09–1.56) and report poor or fair health (OR, 95% CI: 1.62, 1.33–1.97). Subgroup analyses demonstrated attenuation of the higher likelihood of ≥ 5 FVPD among prostate cancer survivors in rural respondents (OR, 95% CI: 0.98, 0.72–1.33). Poorer perceived health was greatest if ≤ 65 years of age (OR, 95% CI: 2.54, 1.79–3.60) and nonsignificant if black (OR, 95% CI: 1.41, 0.70–2.82). Smoking and alcohol which were not significant for the sample as a whole, demonstrated significant associations in certain subgroups.

**Conclusion:**

Although efforts to enhance perceived health and healthy lifestyle behaviors among prostate cancer survivors are warranted, demographic subgroups such as prostate cancer survivors ≤ 65 and rural populations may require more aggressive interventions.

## Background

Prostate cancer is the most common cancer in the US affecting over 200,000 men annually [1]. Over the past 20 years, the survival rate for prostate cancer across all stages has increased to nearly 100% [1] resulting in over 1.8 million prostate cancer survivors nationally [2]. Attention to lifestyle behaviors is an important aspect of quality cancer care for survivors [3]. Cancer survivors are at increased risk for recurrence, secondary cancers, and other chronic diseases such as cardiovascular disease, arthritis, and hypertension that may be attenuated by healthy lifestyle choices [4,5]. Prostate cancer survivors, specifically, may be more apt to die from comorbidities rather than their cancer diagnosis [6,7].

In addition to the potential beneficial impact on comorbidities, healthy lifestyle behaviors may provide other benefits for prostate cancer survivors. Physical activity improves quality of life among prostate cancer survivors [8], reduces treatment related fatigue [8,9], and may slow prostate cancer progression with the potential to reduce mortality [10,11]. A healthy diet may improve physical functioning [12] and slow progression of early stage disease [11]. Although the benefits of smoking cessation among prostate cancer survivors has not been specifically studied, smoking has been associated with an increased risk of developing prostate cancer [13] and with poorer treatment response and survival in cancers other than prostate (e.g., lung, head and neck) [14-16]. Lastly, the benefits of reduced alcohol intake are inadequately studied [17], but higher alcohol intake has been associated with reduced survival in cancers other than prostate (e.g., breast) [18,19].

Although making healthy lifestyle choices is important for cancer survivors, the prevalence of healthy behaviors is less than optimal with prostate cancer survivors being less likely than other cancer survivors (e.g., breast, colorectal) to make healthy changes in their diet and/or physical activity after cancer diagnosis [20-24]. Although inconsistent, the few studies comparing cancer survivors of any type with participants without a cancer diagnosis suggest differences in lifestyle behaviors (e.g., diet, physical activity, smoking, and alcohol) depending on cancer type and age subgroups [3,21,24-28]. The only U.S. study to date comparing prostate cancer survivors specifically with noncancer controls with regard to lifestyle behaviors, noted that prostate cancer survivors reported lower smoking rates (11.1% versus 23.6%) and more frequent moderate-heavy alcohol use (22.3% versus 18.9%) [24]. This study did not adjust for demographic factors or assess diet, body mass index (BMI), and perceived health when assessing differences based on cancer type. Because demographic differences are suspected to occur due to psychosocial, cultural, and economic reasons [29-32], it is fortunate that an Australian population-based study performed such adjustments and confirmed the greater probability of moderate alcohol intake in prostate cancer survivors when compared with non-cancer controls [28]. Although this study did not find differences for other health-related behaviors (including diet and BMI), no assessment of perceived health was reported. Because cancer survivors are at increased risk for poorer perceived health and knowledge of etiolologic factors is inadequate [33], perceived health is of particular importance. A better understanding of how prostate cancer survivors differ from men without prostate cancer and whether these potential differences vary across demographic subgroups will help to focus and prioritize future public health interventions for improving the health and well-being of prostate cancer survivors.

Therefore, our primary study aim was to compare differences in lifestyle behaviors, BMI, and perceived health for men with and without a diagnosis of prostate cancer using a national population-based sample. Our secondary study aim was to explore whether these potential differences in lifestyle, BMI, and perceived health varied across demographic subgroups of age, race, education, and urbanicity. The secondary study aim is particularly important because demographic factors other than age have not been adequately evaluated in related studies.

## Methods

### Study design and participants

Study participants were men, ≥ 40 years of age (n = 63,662) identified from the Behavioral Risk Factor Surveillance System (BRFSS) 2002 data. Details of the BRFSS design [34,35] including its reliability and validity [36] have been described elsewhere, and are briefly described here. The BRFSS is an ongoing national population-based cross-sectional survey conducted in the United States annually since its initiation in 1984. The Centers for Disease Control and Prevention (CDC) collaborates with 54 reporting areas which include all the states, three territories, and the District of Columbia. Trained interviewers use a structured telephone survey of health-related behaviors from noninstitutionalized participants aged 18+ who are identified by the random-digit dialing method. The self-reported information includes demographic data, and other lifestyle factors related to chronic diseases, injuries, and preventable infectious diseases. The BRFSS is approved by the Centers for Disease Control and Prevention Institutional Review Board prior to collection of data.

### Measures

For this study, the main classification variable was a self-reported history of prostate cancer as measured by the following BRFSS question: 'Have you ever been told by a doctor, nurse, or other health professional that you had prostate cancer?' According to CDC protocol, only male participants aged 40+ years old were asked to respond. In our study, men who answered 'yes' were defined as prostate cancer survivors (n = 2,524), while those who answered 'no' were defined as non prostate cancer controls (n = 61,138).

The lifestyle behaviors, BMI, and perceived health were defined as follows. Based on self-report, fruit and vegetable intake was dichotomized as less than five servings per day versus five or more servings per day. Cigarette smoking was dichotomized as current versus nonsmoker. Respondents who reported smoking every day or some days and ≥ 100 cigarettes in their lifetime were classified as current smokers while nonsmokers included both former smokers (i.e., smoked ≥ 100 cigarettes in their lifetime but did not currently smoke) and never smokers. At risk for binge alcohol use was defined as having five or more drinks on one occasion in the past 30 days. Men at risk for heavy alcohol consumption (yes/no) were defined as having an average of more than 2 drinks per day during the preceding month. To assess sedentary behavior (yes/no), respondents reported whether or not they had participated in any leisure-time exercise during the past month, such as running, calisthenics, golf, gardening, or walking. Self-reported weight and height were used to calculate body mass index (BMI; weight [kg]/height [m^2^]) which was then classified into three categories of less than 25 (healthy), 25 to 30 (overweight), more than 30 (obese) for reporting of estimated prevalence percentages. Perceived health was assessed with the following question: "Would you say that in general your health is poor, fair, good, very good, or excellent?" Perceived health was categorized into three levels (i.e., poor/fair, good, and very good/excellent) for analysis.

Because of known associations with lifestyle behaviors in other populations, several potential confounding demographic factors were assessed [37-40]. Self reported age in years was categorized by decade for estimated prevalence percentage reporting only (i.e., the continuous age variable was used in logistic regression analyses). Race was dichotomized into 'black' if the respondent self-reported race as Black or African American, and 'non-black' if White, Asian, Native Hawaiian, or otherPacificIslander, AmericanIndianorAlaskanNative, or otherrace was reported. Education was defined as less than 12 years, 12 years, or more than 12 years of education. Urbanicity was classified as rural versus urban residence based on the Metropolitan Statistical Area (MSA) coding (i.e., rural residence was defined as counties that are not part of a metropolitan statistical area and urban residence was defined as counties in a metropolitan statistical area).

### Data analysis

The weighted 2002 data provided by the CDC were used for all data analyses using SAS statistical software (version 9.1; SAS Institute Inc., Cary, NC, USA). SAS PROC SURVEY routines were used to allow for appropriate weighting procedures necessary to account for the complex sampling strategy and adjust for factors such as non-telephone coverage, non-response, and probability of selection. All of the study measures had minimal missing information (0.1% to 2.0%). Because the annual income had 11% missing data, education level (<1% missing) was chosen as the measure of socioeconomic status. Weighted prevalence estimates of lifestyle behaviors, BMI, perceived health, and demographic factors for prostate cancer survivors and non prostate cancer controls were calculated with differences examined with chi-square test for categorical variables and t-test for continuous variables.

To achieve the primary study aim, the relationships between a history of prostate cancer diagnosis and lifestyle behaviors, BMI, and perceived health were examined among all respondents with unadjusted and adjusted prevalence odds ratios (OR) and 95% confidence intervals (CI) calculated using multivariable logistic regression. Adjustment was made for race, age in years (continuous), education, and urbanicity because these variables have demonstrated the potential for confounding due to an association with lifestyle factors and/or prostate cancer in other populations [13,21,24,37-40]. Because adjustment for additional factors [i.e., health status, whether or not the respondent had a personal doctor, whether or not the respondent had health insurance, geographic region in the U.S. (i.e., west, northeast, south, midwest), and body mass index] did not alter the odds ratios, we have reported only the ORs adjusted for race, age, education, and urbanicity.

To achieve the secondary study aim, the associations between a prostate cancer diagnosis and lifestyle behaviors, BMI, and perceived health were assessed with multivariable logistic regression calculation of adjusted ORs for demographic subgroups. Each demographic characteristic (i.e., age, race, education, and urbanicity) was dichotomized. Only two subgroups were considered for each characteristic to facilitate adequate stratum specific sample sizes. For the subgroup analyses, the ORs were adjusted for the other three demographic characteristics. Specifically, ORs for the age subgroups (i.e., ≤ 65 and > 65 years of age), were adjusted for race, education and urbanicity. Similarly, ORs for race subgroups were adjusted for age, education, and urbanicity; ORs for education subgroups were adjusted for age, race, and urbanicity; and ORs for urbanicity were adjusted for age, race, and education. To test for significant differences in the ORs across demographic strata, each model was repeated with an inclusion of the demographic factor and the appropriate interaction term (i.e., demographic variable*prostate cancer diagnosis).

## Results

Weighted prevalence estimates for demographic factors, lifestyle behaviors, BMI, and perceived health are provided in Table 1. Prostate cancer survivors were older (mean age: 71.7 ± 15.7) compared to non prostate cancer controls (mean age: 55.4 ± 19.7). No significant differences between prostate cancer survivors and non prostate cancer controls were found for race, education level, or urbanicity. The prevalence of all but one lifestyle behavior demonstrated statistically significant differences for prostate cancer survivors versus non prostate cancer controls. Prostate cancer survivors had a higher prevalence of self-reported servings of ≥ five fruit and vegetables servings per day (FVPD) compared with the non prostate cancer controls (30.7% vs. 20.2%) and were more likely to be nonsmokers (88.6% vs. 77.2%). Prostate cancer survivors were significantly less likely to be at risk for binge drinking (6.3% vs. 16.1%) or for heavy alcohol consumption (3.1% vs. 5.4%). No difference in the estimated prevalence of sedentary behavior was noted for prostate cancer survivors and non prostate cancer controls (25.5% versus 24.6%, respectively). Prostate cancer survivors compared with non prostate cancer controls were less likely to perceive their health as very good/excellent (33.0% vs. 50.1%) and to be obese (17.7% vs. 25.0%).

**Table 1:** Weighted prevalence estimates of demographic factors, lifestyle behaviors, body mass index, and perceived health among prostate cancer survivors and noncancer controls, Behavioral Risk Factor Surveillance System, 2002.

	Prostate cancer survivors Percent^†^	Noncancer controls Percent^†^	P value
Age (year)			
40 – 49	2.5%	38.4%	
50 – 59	9.2%	28.7%	
60 – 69	24.1%	18.0%	
70 – 79	43.5%	11.2%	
80 – 89	19.6%	3.4%	
90 +	1.0%	0.3%	<.05
Mean ± SD	71.7 ± 9.8	55.4 ± 11.7	<.05
Race			
Black	9.8%	8.7%	
Non-black	90.2%	91.3%	NS*
Education			
< 12 yrs	14.5%	12.5%	
12 yrs	28.2%	29.1%	
> 12 yrs	57.2%	58.4%	NS
Urbanicity			
Rural	21.5%	22.7%	
Urban	78.5%	77.3%	NS
Fruit and vegetable servings			
≥ 5 per day	30.7%	20.2%	
< 5 per day	69.3%	79.8%	<.05
Smoking status			
Current smoker	11.4%	22.8%	
Non-smoker	88.6%	77.2%	<.05
Binge alcohol use			
At risk	6.3%	16.1%	
Not at risk	93.7%	83.9%	<.05
Heavy alcohol consumption			
At risk	3.1%	5.4%	
Not at risk	96.9%	94.6%	<.05
Sedentary behavior			
Yes	25.5%	24.6%	
No	74.5%	75.4%	NS
Body mass index			
< 25 (normal)	30.2%	27.1%	
25 to 30 (overweight)	52.1%	47.9%	
> 30 (obese)	17.7%	25.0%	<.05
Mean ± SD	26.9 ± 8.8	27.8 ± 9.4	<.05
Perceived health			
Poor/fair	33.6%	19.0%	
Good	33.3%	30.9%	
Very good/excellent	33.0%	50.1%	<.05

^†^All percentages weighted.

*Not significant

For the primary study aim, the adjusted and unadjusted ORs for the associations between history of prostate cancer diagnosis and lifestyle behaviors, BMI, and perceived health are provided in Table 2. Compared with non prostate cancer controls, prostate cancer survivors were no different with regard to prevalence of smoking, binge alcohol risk, heavy alcohol consumption, sedentary, behavior, and obesity but reported a 30% increased likelihood of consuming ≥ 5 FVPD (adjusted OR, 95% CI: 1.30, 1.09 – 1.56). Prostate cancer survivors were also significantly more likely to report poor or fair health with an adjusted 62% increased risk of poor or fair health (adjusted OR, 95% CI: 1.62, 1.33–1.97).

**Table 2:** Adjusted prevalence odds ratio (OR) and 95% confidence interval (CI) for the associations between prostate cancer diagnosis and lifestyle behaviors, body mass index, and perceived health.

Lifestyle/health factor	Unadjusted OR (95% CI)	*Adjusted OR (95% CI)
Fruit and vegetable servings		
≥ 5 per day	1.75 (1.49 – 2.08)	1.30 (1.09 – 1.56)
< 5 per day	¶1.00	¶1.00
Smoking status		
Current smoker	0.44 (0.35 – 0.54)	0.86 (0.68 – 1.08)
Non-smoker	¶1.00	¶1.00
Binge alcohol use		
At risk	0.35 (0.26 – 0.47)	0.81 (0.59 – 1.11)
Not at risk	¶1.00	¶1.00
Heavy alcohol consumption		
At risk	0.57 (0.38 – 0.86)	0.79 (0.50 – 1.24)
Not at risk	¶1.00	¶1.00
Sedentary behavior		
Yes	1.05 (0.90 – 1.23)	0.91 (0.77 – 1.09)
No	¶1.00	¶1.00
Body mass index		
< 25	¶1.00	¶1.00
25 – 30	0.98 (0.82 – 1.16)	1.13 (0.95 – 1.35)
> 30	0.64 (0.52 – 0.78)	0.87 (0.70 – 1.08)
Perceived health		
Poor/fair	2.68 (2.25 – 3.19)	1.62 (1.33 – 1.97)
Good	1.64 (1.37 – 1.95)	1.30 (1.07 – 1.57)
Very good/excellent	¶1.00	¶1.00

*Adjusted for race, age in years, education (< 12 yrs, 12 yrs, > 12 yrs), and urbanicity (rural/urban residence).

¶1.00 – Reference category

For the secondary study aim, adjusted ORs stratified by age, race, education, and urbanicity are provided in Table 3 to explore potential differences in the associations across demographic subgroups between lifestyle behaviors, BMI, and perceived health in prostate cancer survivors and non prostate cancer controls. The increased likelihood of ≥ 5 FVPD among prostate cancer survivors was attenuated in rural respondents with a nonsignificant adjusted OR of 0.98 (95% CI 0.72 – 1.33). The increased likelihood of poor or fair health in prostate cancer survivors was highest for participants ≤ 65 years of age (adjusted OR, 95% CI: 2.54, 1.79 – 3.60), lowest for participants with > 12 years of education (adjusted OR, 95% CI: 1.30, 1.02 – 1.65), and nonsignificant for black respondents (adjusted OR, 95% CI: 1.41, 0.70 – 2.82). Three of the four variables with nonsignificant adjusted ORs prior to stratification (i.e., smoking, binge alcohol risk, and obesity) demonstrated significant associations in certain subgroups after stratification. The lower likelihood of smoking or binge alcohol use in prostate cancer survivors was significant for respondents ≤ 65 years of age only. Sedentary behavior was reported less often by prostate cancer survivors among black respondents and respondents with ≤ 12 years of education. A reduced likelihood of obesity was noted among respondents who were > age 65. Statistical testing of OR differences across strata revealed a greater reduction in heavy alcohol consumption and sedentary behavior among prostate cancer survivors for respondents who were black (p = .038) and reported ≤ 12 years of education (p = .008), respectively. The higher likelihood of prostate cancer survivors to report good rather than very good/excellent health was increased among respondents ≤ 65 years of age (p = .034).

**Table 3:** Adjusted prevalence odds ratio (OR) and 95% confidence interval (CI) for the associations between prostate cancer diagnosis and lifestyle behaviors, body mass index, and perceived health, stratified by age, race, education, and urbanicity.

	Age*		**Race**^†^		**Education**^‡^		**Urbanicity**^§^	
	**≤ 65**	**> 65**	**Black**	**Non-black**	**≤ 12**	**>12**	**Urban**	**Rural**
Fruit/vegetable servings								
≥ 5 per day	1.32 (0.95 – 1.85)	1.35 (1.10 – 1.64)	1.63 (0.89 – 2.99)	1.28 (1.06 – 1.54)	1.37 (0.99 – 1.89)	1.27 (1.03 – 1.56)	1.39 (1.14 – 1.72)	0.98 (0.72 – 1.33)
< 5 per day	¶1.00	¶1.00	¶1.00	¶1.00	¶1.00	¶1.00	¶1.00	¶1.00
**Smoking status**								
**Current smoker**	**0.66** **(0.47 – 0.92)**	**0.88** **(0.64 – 1.21)**	**0.84** **(0.43 – 1.66)**	**0.86** **(0.67 – 1.10)**	**0.96** **(0.70 – 1.32)**	**0.73** **(0.51 – 1.03)**	**0.80** **(0.59 – 1.08)**	**1.06** **(0.78 – 1.45)**
**Non-smoker**	**¶1.00**	**¶1.00**	**¶1.00**	**¶1.00**	**¶1.00**	**¶1.00**	**¶1.00**	**¶1.00**
Binge alcohol use								
At risk	0.56 (0.39 – 0.79)	0.80 (0.51 – 1.28)	0.40 (0.16 – 1.02)	0.85 (0.61 – 1.18)	0.97 (0.67 – 1.40)	0.70 (0.43 – 1.14)	0.77 (0.53 – 1.13)	0.97 (0.62 – 1.50)
Not at risk	¶1.00	¶1.00	¶1.00	¶1.00	¶1.00	¶1.00	¶1.00	¶1.00
**Heavy alcohol consumption**								
**At risk**	**0.65** **(0.34 – 1.22)**	**0.82** **(0.46 – 1.45)**	**0.26**^A^ **(0.06 – 1.02)**	**0.83**^A^ **(0.52 – 1.32)**	**0.68** **(0.36 – 1.27)**	**0.83** **(0.45 – 1.51)**	**0.82** **(0.49 – 1.38)**	**0.62** **(0.30 – 1.27)**
**Not at risk**	**¶1.00**	**¶1.00**	**¶1.00**	**¶1.00**	**¶1.00**	**¶1.00**	**¶1.00**	**¶1.00**
Sedentary behavior								
Yes	1.01 (0.72 – 1.43)	0.93 (0.77 – 1.13)	0.57 (0.34 – 0.95)	0.97 (0.81 – 1.16)	0.78^B ^ (0.61 – 0.99)	1.03^B ^ (0.82 – 1.29)	0.84 (0.68 – 1.05)	1.16 (0.89 – 1.50)
No	¶1.00	¶1.00	¶1.00	¶1.00	¶1.00	¶1.00	¶1.00	¶1.00
**Body mass index**								
**<25**	**¶1.00**	**¶1.00**	**¶1.00**	**¶1.00**	**¶1.00**	**¶1.00**	**¶1.00**	**¶1.00**
**25 – 30**	**1.24** **(0.88 – 1.75)**	**1.12** **(0.91 – 1.37)**	**1.77** **(0.96 – 3.26)**	**1.10** **(0.91 – 1.32)**	**1.27** **(0.92 – 1.74)**	**1.04** **(0.85 – 1.29)**	**1.16** **(0.94 – 1.44)**	**1.04** **(0.80 – 1.35)**
**> 30**	**1.14** **(0.78 – 1.66)**	**0.77** **(0.59 – 1.00)**	**1.30** **(0.69 – 2.45)**	**0.84** **(0.66 – 1.05)**	**0.90** **(0.63 – 1.28)**	**0.85** **(0.65 – 1.11)**	**0.86** **(0.66 – 1.12)**	**0.89** **(0.65 – 1.22)**
Perceived health								
Poor/fair	2.54 (1.79 – 3.60)	1.61 (1.29 – 2.01)	1.41 (0.70 – 2.82)	1.66 (1.35 – 2.03)	1.71 (1.20 – 2.43)	1.30 (1.02 – 1.65)	1.66 (1.31 – 2.10)	1.45 (1.08 – 1.94)
Good	1.82^C ^ (1.32 – 2.50)	1.16^C ^ (0.92 – 1.45)	1.68 (0.85 – 3.31)	1.26 (1.03 – 1.54)	1.11 (0.76 – 1.61)	1.40 (1.13 – 1.74)	1.34 (1.07 – 1.68)	1.12 (0.82 – 1.54)
Very good/excellent	¶1.00	¶1.00	¶1.00	¶1.00	¶1.00	¶1.00	¶1.00	¶1.00

¶1.00 – Reference category; *Adjusted for race, education, and urbanicity; ^†^Adjusted for age, education, and urbanicity; ^‡ ^Adjusted for age, race, and urbanicity; ^§^Adjusted for age, race, and education; ^A ^p value for odds ratios across strata = .038; ^B ^p value for odds ratios across strata = .008; ^C ^p value for odds ratios across strata = .034.

## Discussion

Based on a national, population-based survey, men with a history of prostate cancer do not differ from those without a history of prostate cancer with regard to smoking status, binge and heavy alcohol use, sedentary behavior, and obesity. Prostate cancer survivors may, however, be more apt to consume fruits and vegetables and report poorer perceived health. Demographic characteristics may influence the prevalence of lifestyle behaviors, BMI, and poorer perceived health for prostate cancer survivors compared to those without a history of prostate cancer.

Our study is strengthened by its use of a national, population-based survey and its focus on an understudied cancer survivor population. Our approach was unique in its comparison of prostate cancer survivors with a control population without a prostate cancer diagnosis with regard to lifestyle behaviors, BMI and perceived health. Prior studies have compared lifestyle behaviors among survivors with different cancer types [3,12,20-22] and/or among cancer survivors (all types combined, breast cancer survivors only, or testicular cancer only) and noncancer controls [21,26-28]. However, few studies have used national level population-based surveys [3,21,24,28], only two studies have specifically compared the behaviors of prostate cancer survivors with non-prostate cancer controls [24,28], only one has included comparison of diet and BMI [28], and no prior study has performed a comparison of perceived health. Furthermore, age influences lifestyle behavior prevalence among cancer survivors [21,24] but no prior study has assessed the potential influence of race, education, and urbanicity on the prevalence of health behaviors, BMI, and perceived health among prostate cancer survivors when compared with controls.

With regard to lifestyle behaviors, the higher fruit and vegetable consummation reported by prostate cancer survivors is consistent with prior studies and may be partially explained by the fact that a cancer diagnosis may cause an individual to adopt healthier behaviors [25,41]. However, the cross-sectional study design and lack of information on time since diagnosis in the BRFSS data precludes proof of this potential cause and effect relationship. The cross-sectional design also introduces the possibility of survival bias. Because obesity has been associated with increased prostate cancer recurrence and mortality [42-44] and may play a role in the increased prostate cancer risk in black men [42], survival bias could bias the prevalence odds ratios to reflect better diet and activity behaviors among the prostate cancer survivors. Therefore, the significant reduction in prevalence of consuming < 5 daily servings of fruits and vegetables and sedentary behavior (especially among black men) is consistent with the association between obesity and prostate cancer risk. This finding is also consistent with a recent study demonstrating that a lifestyle intervention including diet, exercise, and stress management may reduce prostate cancer progression [11].

The most prevalent poor lifestyle behavior was inadequate fruit and vegetable intake followed by sedentary behavior. Because the 2002 BRFSS dataset utilized for this study categorized fruit and vegetable intake according to the recommendations at that time of ≥ 5 servings per day, it would be expected that compliance with the more recent recommendation of ≥ 9 fruit and vegetable servings daily [45] would be even lower. Smoking and binge/heavy alcohol use, although present, were less frequent. Our finding that 69.3% of prostate cancer survivors reported eating < 5 daily servings of fruits and vegetables is consistent with that reported by Demark-Wahnefried [20] (65%) but slightly higher than that reported by Coups [21] (i.e., 39.8%). The prevalence of smoking and alcohol was also consistent with prior studies [20,21,24]. Because we did not assess regular physical activity but only sedentary behavior, comparison with prior studies is difficult. Nevertheless, our results are consistent with prior reports of regular exercise among 29.3% [21] to 62% [20] of prostate cancer survivors.

Although measurement bias may exist because lifestyle behaviors and prostate cancer diagnosis were self-reported, it is reassuring that our lifestyle prevalence rates were consistent with other studies and prostate cancer diagnosis leads to sufficient physician attention and treatment that it is less likely that the diagnosis would be unknown or forgotten. If respondents had been asked to recall lifestyle exposures prior to cancer diagnosis, those with cancer might over or underestimate the behaviors. However, respondents were asked about current behavior suggesting that self-report would be expected to be similar in both the prostate cancer survivors and the comparison group and, therefore, would not significantly influence the reported associations. Also, residual confounding may exist when analyzing nonrandomized, observational study using public domain data with predetermined variables and data collection procedures as with the BRFSS data, but adjustment for surrogate markers for health-related confounders did not significantly change the odds ratios and the increased generalizability achieved with a national sample outweighs this potential risk. Additional caution is advised due to the possibility of statistical associations by chance alone when multiple comparisons are performed (as in Table 3) and reduced precision of the odds ratios due to the large age difference between those with and without a prostate cancer diagnosis. Moreover, our analyses are limited by the lack of information about primary cancer diagnoses other than prostate cancer. It is feasible that the patients without a prostate cancer diagnosis had a cancer diagnosis of another type and/or the prostate cancer patients had more than one cancer diagnoses. Because of the increased risk of second primary cancers after prostate cancer treatment [46], it is possible that differences reported for the prostate cancer patients are primarily related to greater overall cancer burden rather than prostate cancer specifically. Nevertheless, confirmation of our study results in populations with documentation of prostate cancer diagnosis based on other sources besides self-report (e.g., cancer registries) and follow-up studies testing the hypotheses generated by our study are warranted. Such studies should assess the potential confounding or moderating effect of other cancer diagnoses on associations reported.

The suboptimal practice of healthful lifestyle behaviors is especially concerning among cancer survivors because they often report poorer health [24,33], a finding also noted for the prostate cancer survivors in our study. This lower perceived health may be related to a negative psychological impact of a cancer diagnosis and/or higher prevalence of chronic comorbid disease other than cancer [4,5]. Prostate cancer survivors who make healthier lifestyle choices report better quality of life, suggesting that addressing these inadequacies may be a model for improving poor perceived health [47].

## Conclusion

Our results suggest that efforts to enhance perceived health and healthy lifestyle behaviors among prostate cancer survivors are warranted. Certain demographic subgroups may warrant more aggressive interventions such as addressing the higher odds for poorer perceived health in prostate cancer survivors ≤ 65 and lower odds for fruits and vegetable intake in rural populations. Moreover, examining the reasons for the reduced at risk alcohol consumption in black respondents and less prevalent sedentary behavior in respondents with ≤ 12 years of education, may suggest cultural influences that could be used in other lifestyle interventions or sources of measurement error in certain subgroups (e.g., less educated individuals may report occupational physical activity even when self-reported leisure activity is requested). Prospective, cohort studies are needed to assess the changes in lifestyle behaviors, BMI, and perceived health that occur after a prostate cancer diagnosis. These studies should include diverse study populations with regard to age, race, education, and geographic location to confirm potential differences across demographic strata with regard to lifestyle behaviors, BMI, and perceived health. Outcome measures should be designed to explore the potential reasons for differences across demographic strata [e.g., socio-cultural, social cognitive (e.g., readiness for change)]. Discovering these reasons will guide the design of effective behavior change interventions to improve lifestyle behaviors which in turn has the potential to improve the quality of life, health, and survival of prostate cancer survivors.

## Competing interests

The author(s) declare that they have no competing interests.

## Authors' contributions

LR conceived the study, participated in study design and coordination, and wrote a substantial portion of the manuscript. KC contributed to refinement of the study design and analyses and participated in manuscript preparation. RG performed a portion of the data analysis and assisted in manuscript writing and revision. SM performed the remaining portion of the data analysis, contributed to refinement of study design and analysis plan, assisted with data presentation, and provided manuscript preparation assistance. RI contributed to refinement of the study design and analyses and participated in manuscript preparation. All authors have read and approved the final manuscript.

## Pre-publication history

The pre-publication history for this paper can be accessed here:


